# Recent Advances in the Science of Light

**Published:** 1865-01

**Authors:** J. S. Jewell

**Affiliations:** Prof. Anatomy in Chicago Medical College


### ARTICLE II.

### RECENT ADVANCES IN THE SCIENCE OF LIGHT.

BY J. S. JEWELL, M.D., Prof. Anatomy in Chicago Medical College.

The advance from a material to a phenominal or wave theory of light, was perhaps the most signal in the history of optics. In this respect the science of light has kept pace with the new views and generalizations which have from time to time prevailed, concerning force and motion. The admission that light is not a kind of matter but a kind of motion, opened up a new field for, and gave a new direction, to research.

Some of the advances, in the past few years, of our knowledge of the nature and relations of light are so novel and seem to open such a promising field to chemistry and physics, and are likely to prove of such signal practical value to the chemist and physicist, that it may not be improper, in a short article, to collect and exhibit briefly the results of modern research in this direction. In order to do this intelligibly, it will be needful to go back a little and notice the successive steps in the march of discovery. The account I shall subjoin is taken from various sources, as from notices and papers in scientific works, periodicals and foreign reviews, &c.:—

In 1675, NEWTON presented to the Royal Society his memorable treatise on optics. In this paper was detailed his discovery of the solar spectrum, and the singular fact of the compound nature of light, and the colored bands which, in invariable order, traverse the spectrum.

The spectrum was reëxamined by that singularly ingenious experimenter, Dr. WOLLISTON. "It was in 1802, that he, by preventing the overlapping of the colored bands so they should not interfere with each other, discovered that great pecularity in solar light, which in later times has led to such astounding discoveries in the sun itself." He observed when light was passed through a narrow slit and afterwards permitted to fall on the prism, that the spectrum produced was traversed by certain dark bands, of which WOLLISTON counted only seven. The bands seemed as shadows on an otherwise luminous background.

It is, however, to the great German optician, FRAUNHOFER, we are indebted for the first careful examination of these lines. By superior arrangements, he was enabled to discover a far greater number than HOLLASTON had. He counted no less than 500 between the violet and the red, in the spectrum, and in 1815, produced a beautiful map of them. The principal lines he designated by the letters of the alphabet, hence the expression Fraunhofer's line, A, D, &c.

FRAUNHOFER made careful measurements of these lines, and found them invariably to occupy the same relative position, whether he employed direct solar light, or reflected light from the Moon or Venus. But he observed this singular fact:—In the spectra of the fixed stars, the lines did not coincide with those observed in the solar spectrum. At this early period he reached the conclusion, that the cause for this change in the spectra must be caused by, and reveal some difference in, the constitution of the bodies remitting the light. But the discovery of the wonderful import of these lines was reserved for others.

This branch of science was much advanced by the labors of HERSCHEL, who was able to distinguish some of the metals by their spectra. In 1826, TALBOT, from an examination of certain parts of the spectra of several metals, was led to express an opinion that "if this difference of spectra should be found to be correct, and applicable to the other rays, a glance at the prismatic spectrum of a flame may show it to contain substances which it would otherwise require a laborious chemical analysis to effect." In 1834, he described the spectra of strontium and lithium, and declared his ability, by this means, to detect the minutest portions of these substances.

Prof. STOKES, in his admirable reseaches on fluorescence, showed that similar dark lines exist beyond the violet, requiring special arrangements to render them visible. Some 2000 of these dark lines have been mapped by Sir DAVID BREWSTER and Dr. GLADSTONE, in that part of the spectrum between the red and violet.

This line of research was conducted still farther by Mr. W. H. MILLER, DANIEL and Prof. WHEATSONE. Prof. DRAPER, between the years of 1843 and 1848 published a series of papers on this subject, in which was detailed the results of his own valuable researches.

That both simple and compound bodies may give lines in the spectrum, and that two simple substances which do not singly produce them may in their compounds, and that the vapors of simple substances may exhibit lines while they disappear in their compounds, together with other facts, were shown in 1845, by

W. A. MILLER. FOUCAULT, in 1849, observed when he permitted sunlight to fall on the voltaic ark, and then viewed this compound light through a prism, that the usual double brilliant line of the electric light corresponded exactly with the double black line in solar light, and when they were permitted to overlap the dark solar lines unexpectedly became more distinct as dark lines.

Though these facts, with others of like kind, materially advanced our knowledge of the spectrum, they constitute only the prelude to the most important researches in their bearings on the progress of physical and chemical science which have been made perhaps in modern times.

The Prof. of physics at the University of Heidelberg, taking up the thread where it had been dropped by earlier observers, has been conducted to the most singular and beautiful discoveries.

The labors of MASSON, who examined the spectra of various metals employed as dischargers to Leyden jars, and of AUGSTROM, who showed that from the intense heat of the electric current he was able to discern two spectra—one due to the metal, the other to the atmosphere itself, and of VAN. DER. WILLINGER, and MAXWELL, the latter of whom undertook experiments to determine the wave lengths of different kinds of light, and also of PLUCKER, who examined the spectra of the electric current in rarified gases and vapors, should be considered as contributing to the signal advance in this department of science, in which KIRCHOFF, and his coworker BUNSON, have rendered their names immortal, a short account of which will follow.

The instrument employed by these observers, consisted of several prisms of the finest workmanship, arranged in particular order one after another, by which means they obtain great dispersive power, and were enabled to magnify to a very great extent the primary spectrum, the lines were examined by means of a telescope, several feet distant, magnifying about 40 times, the whole instrument was arranged with admirable precision and revealed spectral phenomena, to the beauty of which there is said to be scarcely a parallel.

The purpose of KIRCHOFF was to measure the absolute and relative width of the dark lines, and intervals between the lines crossing the spectrum; also to determine the depth or range of shade or color, and faithfully to delineate them on a map which he set himself to produce.

In all the respects named, the lines were found to differ in different parts of the spectrum—in width and in intensity of color. The measurements were made by cross wires in the instrument, and with surpassing accuracy. The shades representing the lines were laid on the maps in India ink, with almost infinite trouble, as we may well suppose, when we learn that though years were employed in its construction, it only includes about one-third of the spectrum between the violet and red, and entailed the almost total loss of vision in the indefatigable observer.

But before we shall be able to understand how the solar spectrum can teach us anything of the chemical constitution of the solar atmosphere, we must look in another direction a few moments, and see how the constitution of terrestrial matter can be determined by an examination of the light different kinds of matter, when heated, emits.

That different kinds of matter, when heated, emit light of peculiar kinds, has been long known. This fact has been applied in many ways to pyrotechny; and the tint communicated to the blowpipe flame has been employed as a means of distinguishing substances one from another. This fact, until recently, was known to hold good for only a few substances, but is now known, or believed, to hold good for all elementary substances. This fact is made the basis of one of the most beautiful and useful additions to chemistry, of modern times, viz.: spectrum analysis. This kind of analysis is made independently of the ordinary appliances of chemistry, It only requires that the substance to be examined shall be in a state of luminous gas or vapor, that it may emit its peculiar light. That it is possible to bring all substances into this state is a matter of experiment. The ways in which this is accomplished are various, but for most substances it is only necessary to place a small portion of one of their readily decomposable compounds in the flame of a spirit lamp or a gas jet. The compound volatilizes, and while in the gaseous state is heated in the flame till luminous. But other means for the metals fused with difficulty, and for the permanent gases, is necessary, and will be alluded to as we proceed.

Now, the fact that the light emitted from various substances is different, cannot usually be perceived by the eye alone. To determine this we must examine their spectra. The same kind of instrument will be required for this, as already referred to, for exhibition of the solar spectrum. There only is it, that each substance writes down the unalterable law of its relations to matter in the brilliant language of the spectrum.

To examine the spectra of all chemical elements, was a work begun several years since by BUNSEN and KIRCHOFF, and the results which they have obtained bid fair to revolutionize, in important respects, analytical chemistry.

All who have paid the slightest attention to qualitative analysis, will remember what a bewildering series of tests, re-agents, solutions, &c., are frequently required to establish the identity of a substance, but in the analysis of their spectra, the case is entirely different.

The instrument being arranged, we place before it the flame of a lamp or burner, and in it place one of the easily decomposable compounds of a metal, say cloride of sodium, and then with the telescope observe the spectrum. In the case supposed we should find a couple of bright yellow lines close together, and the remainder of the spectrum perfectly dark, this is the invariable appearence of sodium, and so minute is the quantity required as to appear incredible, if the delicacy of this test was not shown by experiment. So minute a quantity as the 180,000,000,000th of a grain will yield the characteristic spectrum. After reading this we will learn without any surprise, it has been found that common salt exists in a finely divided state all through the air. This statement, taken with other collateral ones, suggest some interesting reflections, as to the possible floating contents of the atmosphere. But suppose we introduce into the flame a potassium compound. The flame will instantly acquire a purple tint, and the spectrum will be observed as a central white portion, bounded at one end by a violet band and at the other by a red band. So too with all the metals, they have their peculiar spectra, invariable under the same conditions, and the person acquainted with their characteristic appearances can instantly recognize them. Of the surpassing beauty of the phenomena of this branch of science, no adequate conception can be formed it is said.

Prof. BUNSEN thus describes what he witnessed when he had placed a mixture of many metals in the flame:—"I took a mixture consisting of the chloride of lithium, chloride of sodium, calcium, strontium, barium, &c., containing at most the $\frac{1}{1000}$th of a grain of each substance. This mixture I placed in the flame and observed the result. First, the intense yellow sodium lines appeared on a background of pale continuous spectrum. As these began to be less distinct, the pale potassium lines were seen, and then the red lithium line came out, whilst the barium lines came out in all their vividness. The sodium, lithium, potassium, and barium salts were now almost volatilized, and after a few moments the strontium and calcium lines came out as from a dissolving view, gradually attaining their characteristic brightness and form."

If the foregoing remarks be true, it will be unnecessary to show what a remarkable service has been done analytical chemistry. But it is not here alone that its utility ends. As a means of discovery, it will probably prove to be the most efficient ever employed.

In examining the spectra of alkalies contained in certain mineral waters obtained at Durckheim, in the Palatinate, he observed some lines not like any he had seen before. Convinced they must be due to the presence of some new element, he began immediately the evaporation of 40 tons of the water. In subsequent examinations he discovered fresh signs of perturbation in the spectrum, and at last was able, from a vast quantity of water to obtain a sufficient amount of two new metals for examination, which he named respectively, cesium and rubidium, the former on account of its bluish gray color, and the latter because of its dark red color. They are distinguished in their spectra, the one by splendid violet, and the other by deep red lines. Singularly enough neither of these metals can be said to be very rare, especially the latter, which may now be obtained by the pound, and is found copiously in lepidolite. Since that time another new metal has been, by similar means, discovered by Mr. Crookes. It is called thallium, and the spectrum of which is distinguished by one bright green band. It will be impossible to carry out these facts to their obvious consequences, but it may readily be perceived of what surpassing interest this is to be as a means of discovery, and as the most important acquisition ever made perhaps to analytical chemistry.

When we reflect on the probable future revelations, this line of research is likely to give us, of the intimate constitution of matter, and of light itself, and other correlative phenomena, as exhibited in these relations, we can scarcely be too sanguine in our expectations.

Let us now turn our attention, for a few moments, to another application of the facts above detailed, perhaps the most astonishing of all. Fraunhofer, so early as 1815, observed that the two lines now known to be sodium lines corresponded in refrangibility with the two lines in the solar spectrum known as Fraunhofer's line D; and the same fact was observed by Brewster, in regard to the correspondence between the potassium lines and the solar lines A. In these two cases, as well as in others, by subsequent observers, there was found to be a complete coincidence between the metallic spectra and certain parts of the solar spectrum except in color.

To test this coincidence, Kirchoff performed the following remarkable experiments, and in which we will find the facts which lie at the foundation of solar chemistry:—"In order to test this" says he, "I obtained a tolerable bright solar spectrum, and brought a flame colored by sodium vapor in front of the slit, I then saw the dark lines D change into bright ones. The flame of Bunson's lamp threw the bright sodium lines upon the solar spectrum, with unexpected brilliancy. In order to find out the extent to which the intensity of the solar spectrum could be increased, without impairing the distinctnesss of the sodium lines, I allowed the full sunlight to fall through the sodium flame, and to my astonishment I saw that the dark lines D appeared with an extraordinary degree of clearness. I then exchanged the sunlight, for the Drummond or oxyhydrogen lime light, which like that of all incandescent solid and liquid bodies give spectra containing no dark lines. When this light was permitted to fall through a suitable flame colored by common salt, dark lines were seen in the spectrum, in the position of the sodium lines. The same phenomenon was observed if instead of the heated lime, a platinum wire was used, which being heated in a flame was brought to a temperature near the melting point by passing an electric current through it. The phenomenon is easily explained, on the supposition that the sodium flame absorbs rays of the same degree of refrangibility as those it emits while it is transparent to all other rays." Thus he succeeded in producing artificial sunlight, as far as producing one of Fraunhofer's lines is concerned.

Now, several facts are to be noted from these experiments:—

1st. The sodium lines correspond perfectly, except in color, with a certain part of the solar spectrum.

2d. By permitting sunlight to fall through the sodium flame the distinctive color of the sodium lines disappeared entirely, and the dark lines of the solar spectrum alone appeared.

3d. The same result was secured if the Drummond light was employed instead of solar light; and in this way there was a part of its spectrum produced with the utmost exactness, though it is well known, all incandescent, solid and liquid, bodies give spectra without dark lines.

4th. That when light is transmitted through any substance, in a vaporous or gaseous state, while luminous with heat, it absorbs and arrests, that kind of light it emits with a completeness in proportion to its intensity; so that the lines that appear in the spectrum are dark, and, therefore, not characteristically colored.

5th. These dark lines are found to correspond to the dark lines in that part of the solar spectrum when the rays of the given color are known to be placed relatively from their observed refrangibility.

It thus appears many parts of the solar spectrum may be artificially produced, and in the completest manner; and that we can determine the conditions upon which these facts depend; and if so, may be prepared to explain some of the spectral phenomena of solar light on the same grounds.

The analysis of spectra of the metals has furnished MILSCHELICH some new facts. He finds metals do not yield a spectrum in all their compounds, and that they do not give the same in different compounds. The character of the spectrum was found to depend upon whether it was produced by the metal or one of its compounds of the first order. The compound may have a spectrum different from the metal from which it is derived. He performed some interesting experiments in the way of determining the temperature at which compounds are decomposed, whereby they may yield the characteristic spectra of their elements; and on the facts thus elicited, bases some interesting speculations as to the temperature of the sun or its atmosphere.

We must not omit to mention a law announced by KIRCHOFF, which is denominated the "law of exchange," which asserts that the relation between the amount of heat or of light emitted from all bodies is invariable for the same temperature. A similar result was also arrived at by other observers.

KIRCHOFF now set himself to map the electric spectra of the metals, and, as his guide, chose the dark lines of the solar spectrum, and was surprised to find a complete coincidence between the bright iron lines and certain dark solar lines. Just as the sodium and potassium lines found corresponding lines at D and A, among the lines of the solar spectrum. The utmost pains were taken to verify this. The coincidence was too remarkable to be the effect of chance. Now, the only difference in the spectra was in the *color* of the lines. In order to explain these phenomena, we must now recur to the results of some experiments recorded above. We may suppose the light of the sun, after its emission, to have passed through the vapor of iron in the solar atmosphere. Now, we have just seen where a flame is colored with sodium vapor, and the lime light was passed through the flame, how the yellow sodium lines became dark, giving exactly the Fraunhofer line D, in the solar spectrum; and just so if we substitute iron for the sodium in the flame, we will have, instead of the red lines in the iron spectrum, dark ones, which now correspond in all particulars with that part of the solar spectrum with which it was first composed. We have, in fact, reproduced part of the solar spectrum, with the utmost fidelity.

Now, the only chance for mistake, in this case, is in this:—that the iron and other metals affecting the solar light may be in our own atmosphere, rather than in the solar atmosphere. But then we do not have the necessary temperature in our own air to vaporize such elements. Then, if this modification of light was made in our own atmosphere, we should expect the light from the stars and the solar light to correspond. They do not, however, correspond nor do the stars always correspond in their spectra. We have not time to examine all the particulars upon which this marvellous conclusion rests; but the evidence, when thoroughly examined, will be found to establish the fact of the existence of iron and many other elementary substances on the sun, as clearly as any fact in physical science. The induction, through which this conclusion has been reached, is eminently satisfactory.

Now, an examination has been undertaken concerning the light of the fixed stars, and we may expect the most novel and interesting results. This branch of science has been styled "solar" or "stellar chemistry," and has now engaged the attention of the first astronomers and physicists, both in Europe and this country. The astronomer royal of England, Prof. AIRY, SECCHI and DONATE on the continent, and RUTHERFORD in this country, are engaged in the exploration of this brilliant path of research. Thus it will be seen, that we have in this new branch of science, the most valuable acquisition ever made to analytical chemistry, and, at the same time, the most efficient means for enlarging the field of chemical and physical science. There is no greater triumph of science than the key it has given us by which to translate the mysterious though beautiful language of the spectrum; and if anything more is to be revealed of the intimate constitution of matter, or of those singular movements or force of matter known as light, heat, and electricity, and their correlations, it will, in all probability, be through this remarkable way.

